# A Feasibility Study of Splintage by 3D Scanning and Printing: Process and Evaluation of Current 3D Printing Material

**DOI:** 10.3390/ma19061146

**Published:** 2026-03-15

**Authors:** Sze Wai Anson Li, Sze Wa Afra Mok, Sze Wing Wong, Bohao Yang, Jackie Ngai-Man Chan, Kenneth N. K. Fong, Sam Chi Chung Chan, Chung Man Joy Lau, Benson Wui-Man Lau

**Affiliations:** Department of Rehabilitation Sciences, The Hong Kong Polytechnic University, Hong Kong, China; cwai8866@gmail.com (S.W.A.L.); aframok.sw@gmail.com (S.W.A.M.); wswconnection@gmail.com (S.W.W.); peterbohao.yang@connect.polyu.hk (B.Y.); jackieman.chan@connect.polyu.hk (J.N.-M.C.); rsnkfong@polyu.edu.hk (K.N.K.F.); samcc.chan@polyu.edu.hk (S.C.C.C.); joy.lau@polyu.edu.hk (C.M.J.L.)

**Keywords:** 3D printing technology, 3D-printed splint, splint fabrication, rehabilitation technology, occupational therapy

## Abstract

Three-dimensional (3D) scanning and printing technologies enable the production of personalized rehabilitation splints, yet challenges such as scanning artifacts in complex anatomical areas (e.g., finger webs), lengthy post-processing, long printing times, and material limitations (e.g., brittleness and poor breathability) hinder routine clinical adoption. This feasibility study developed and evaluated a clinician-accessible protocol for fabricating cock-up wrist splints using 3D scanning (Creaform GO!SCAN 50 with VXelements 4.1), modeling (Materialise Magics), and fused deposition modeling printing with polylactic acid (PLA) on a MakerBot Replicator+. Five healthy participants wore the splints for one week, with user satisfaction assessed via the Quebec User Evaluation of Satisfaction with Assistive Technology (QUEST 2.0; average total score 4.14/5, range 3.75–4.42) questionnaire. An experienced occupational therapist provided expert feedback. High satisfaction was reported for weight (4.6/5) and ease of use (4.6/5), confirming advantages over traditional thermoplastic splints in lightness and esthetics. However, lower scores for durability (3.6/5), comfort (3.6/5), and effectiveness (3.6/5) stemmed from PLA brittleness (cracking under load or overtightening), rough surfaces despite vapor polishing, inadequate ventilation causing moisture buildup, and fit issues (e.g., pressure points). Printing time averaged 9–19 h per splint. The protocol demonstrates proof-of-concept feasibility for clinicians with basic computer techniques, but material constraints and process refinements are required for reliable application in patient populations.

## 1. Introduction

Splintage is one of the intervention modalities prescribed to patients in rehabilitation. It facilitates a patient’s recovery by protecting the injured body part, allowing early mobilization, correcting the alignment and supporting the body part while working [1,2]. The conventional approach of fabrication is performed by a clinician who is responsible for designing, processing, molding, post-processing and fitting the thermoplastic splint for the patient. As the process is customized and the splint is tailor-made, it is demanding in terms of the expertise of the therapist and time for fabrication [3].

Three-dimensional (3D) scanning, modeling using computer-aided design (CAD) and printing have been applied to fabricate 3D-printed splints in rehabilitation for the last decade in light of the availability of affordable equipment [3]. The 3D-printed splint is found to be effective in the management of various medical conditions, such as improving hand function and activities of daily living (ADLs) for patients with forearm fractures and reduction in spasticity in stroke patients [4,5].

The 3D-printed splint is also suggested to have several advantages over the thermoplastic splint produced via the conventional approach. The 3D-printed splint is lighter and has better esthetic appearance. It involves shorter production time and faster morphology acquisition, while patients have a more pleasant experience [3,6,7]. Anatomical data captured by 3D scanning contributes to a more precise and ergonomic splint that fits the topography of the patient’s body parts [8].

However, there are still some concerns in applying these technologies in clinical settings. For instance, undesirable scanning artifacts (e.g., holes) in the scanned model are commonly seen in areas that are unreachable by the scanning light rays of the 3D scanner, such as the folds in the finger webs [9]. This could greatly affect the subsequent procedures. In addition, choice of appropriate scanner and printer, technique to scan a large body area, post-processing of printed splints and parameter settings for scanning and printing are challenges when applying the 3D technologies in clinical situation [9]. In order to ensure the quality of the splint, clinicians are required to be familiar with the 3D scanning and printing hardware, procedures and software.

As many studies have suggested, complicated technology for splint production is one of the major barriers to the application of the 3D-printed splint [5,10,11]. Technical skills of utilizing computer programs, 3D scanning and printing are required to fabricate individualized 3D-printed splints for promoting users’ compliance to wearing the splint [5,12]. It is proposed that the challenging splint design process using software has hindered the application of 3D scanning and printing technologies to splint production in hospitals [13]. As these technologies could be a new field to the clinicians, it seems that abundant time and resources should be invested in equipping technicians with the relevant knowledge and skills.

Having limited in-house expertise of 3D scanning and printing technologies, some clinicians have attempted to use prototypes (3D models of splints) available online. However, the quality of prototypes acquired online is uncertain and they cannot be tailor-made for patients, since the prototype might be developed or reviewed by engineers rather than experienced clinicians [11,12]. Although there are some software-based tools that claim to be able to generate prototype designs of 3D-printed splints automatically, the construction method is usually unclear [14]. Clinicians may need to assess each prototype individually to guarantee the quality of design. Much time and effort are needed to search for and identify suitable prototypes while bearing the risk that no appropriate prototype is found. Indeed, enabling clinicians to produce 3D-printed splints would be the ideal solution to safeguard the quality. One study proposes that clinicians could have an active role in the splint design process [6]. Meanwhile, another study suggests that support could be offered to people with limited technical experience to handle the fabrication process [15]. Therefore, a splint guideline for building up the technical competency in 3D-printed splint production might contribute to a more efficient learning process, which in turn promotes the application of 3D-printed splints.

Recent studies have explored PLA for 3D-printed orthoses, highlighting its tensile strength (~50–70 MPa) and rigidity suitable for short-term immobilization, but noting brittleness and limited elasticity compared to alternatives like PETG or TPU, which offer better flexibility and fatigue resistance [16,17,18]. These material properties, combined with workflow complexities (e.g., software navigation, calibration needs), remain barriers to widespread adoption in clinical settings. This study builds on these findings by testing a simplified, step-by-step protocol requiring only basic computer literacy (e.g., following guided software steps without advanced CAD expertise) to enable occupational therapists to produce individualized cock-up splints independently. The premise is to bridge the gap between emerging technology and practical rehabilitation use, providing preliminary insights into process viability, user/expert feedback, and PLA limitations in a controlled feasibility context.

This study aims to explore a practical approach to using 3D scanning and printing technologies to fabricate hand splints as well as offering a splint manual for clinicians who are interested in adopting these technologies to produce 3D-printed splints in their practice. It could facilitate manual users to learn and apply the CAD skills and enable 3D-printed splints to be more applicable in clinical settings.

## 2. Methods

### 2.1. Overall Procedure and Specifications of Materials

The wrist splints made for the participants were customized to offer certain functionality and support. About two-thirds of the participant’s forearm was covered by the splint, providing sufficient stability while preserving some degree of mobility. The U-shaped splints were made to fit around half of the forearm, providing an appropriate fit without being unduly confined. To support the wrist and hand, they took a volar approach, which places them on the palm side of the forearm. To ensure that participants could maintain a functional range of motion, the splints were designed to provide wrist extension of at least 20 degrees. Furthermore, while wearing the splints, they allowed flexion at the metacarpophalangeal joints (MPJs), which allowed finger movement and simplified hand functions. Support, comfort, and functionality were all harmonized in this design to satisfy the needs of the study. This proof-of-concept feasibility study focused on key aspects including acceptability (user satisfaction via QUEST 2.0), practicality (process times, clinician accessibility), and limited efficacy (preliminary performance observations), following frameworks for early-stage rehabilitation technology evaluation [19,20,21]. No strict predefined quantitative success criteria were set, as the emphasis was on process exploration and identification of barriers for future optimization.

**Figure 1:**
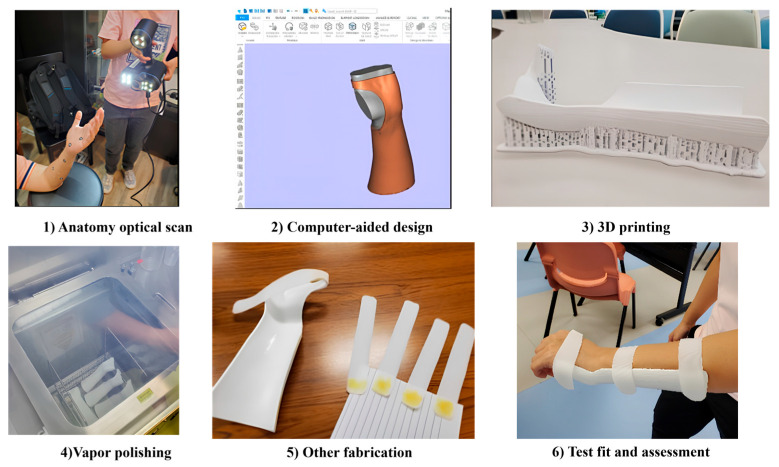
Workflow for 3D-printed wrist splint production.

The feasibility study of 3D scanning splintage involves a multi-step process to design and fabricate a functional splint. This section presents the operational guidelines to tasks involved in the 3D scanning, sketching and 3D printing stages. The overall process is depicted in Figure 1. First, to ensure an exact portrayal of the patient’s distinct characteristics, an optical scan of the anatomy is conducted to obtain precise 3D data of the affected area. Following the input of this scanned data into computer-aided design software, the splint is digitally created to satisfy the patient’s unique needs. After the design is complete, a physical prototype is produced by 3D printing it with the proper materials. Vapor polishing is applied to the printed splint to smooth out the surface and increase patient comfort. To further tailor the splint or add functional elements as needed, additional fabrication procedures may be required. The patient is then given a test fit for the splint, and its performance and fitness are carefully evaluated to make sure it satisfies functional and clinical requirements.

In terms of the specification of the 3D scanner and its software, the 3D printer and materials for 3D printing utilized in this study are listed in Table 1. To guarantee accuracy and functionality, particular materials and equipment were used in the 3D scanning and production process. The VXelements 4.1 software and the Creaform GO!SCAN 50, both created by Creaform (MakerBot Industries, Brooklyn, NY, USA), were used to scan the anatomical surface. Materialise’s Magic^®^ program (Materialise, Leuven, Belgium, website: https://www.materialise.com/en), an effective tool for processing and fine-tuning the scanned data, was used to produce the 3D design of the splint. A MakerBot Replicator+ 3D printer (MakerBot Industries, Brooklyn, NY, USA) with a Smart Extruder+ was used for fabrication, and fused deposition modeling (FDM) was used to create the splint. To guarantee mechanical accuracy and a sturdy construction, a linear pattern and a layer thickness of 0.2 mm were printed using polylactic acid (PLA), a lightweight and biodegradable substance.

### 2.2. 3D Scanning

**Figure 2:**
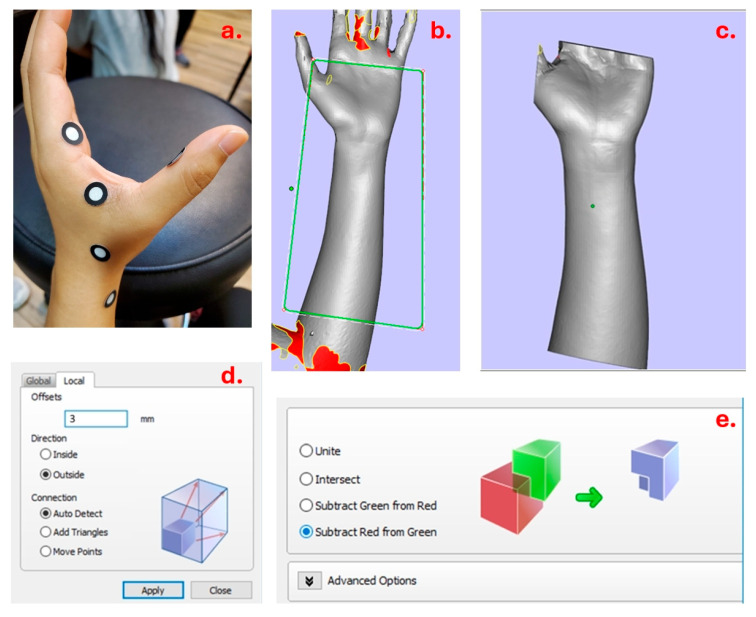
3D modeling. (**a**) Attaching targets to thumb web space; (**b**,**c**) selecting the specific area to design and plotting the general outline; (**d**) duplicate model is adjusted to be 3 mm larger than the original, effectively reflecting the required splint thickness; (**e**) removing the original model from the duplicated model and forming a hollow structure inside the 3D model.

The Creaform GO!SCAN 50 scanner and Creaform VXelements 4.1 were the 3D scanner and software used to scan the participant’s forearm and hand, respectively. This scanner model was suitable for hands and forearms, as these body parts fell within the part size range of 0.3–3.0 m. It also had a resolution of 0.500 mm for scanning body parts in sufficient detail. First, the scanner was calibrated using a calibration plate in accordance with the instructions of manufacturer. The skin of the hands and forearm was then covered with adhesive positioning targets to aid in the scanning process (Figure 2a), especially in regions like the finger web that were not as accessible to the scanner’s laser beams. The individual was then properly positioned, sitting next to a table with the wrist extended at a 20-degree angle, the forearm supinated, and the non-dominant elbow flexed and supported by the table, all while keeping the palm gesture open. There was no set starting point or need for instruction when the operator utilized the scanner to capture the hand and forearm. In order to prevent eye irritation from the light beam of the scanner, patients were told to close their eyes while maintaining the stance in order to shield their eyes. After finishing, the scanned model file was exported in OBJ format so that Materialise Magics could process it further.

### 2.3. 3D Sketching/Modeling

Materialise Magics was adopted for 3D modeling. The scanned model was first imported into the software, and it is likely to have regions that are not well scanned and are presented as missing parts in the raw imported model. In this way, by using Materialise Magics (Materialise, Leuven, Belgium), the model was then modified to correct and fill the missing regions in the surface that were not captured in good form during the 3D scanning process. After reconstructing the surface of the scanned model, the model was ready for building a template of the hand splint (Figure 2b,c). The distal palmar crease and thenar crease of the hand and the styloid process of ulna were used as reference points to guide the trimming process, to avoid potential pressure points and allow finger and thumb movements while wearing the 3D-printed splint. To achieve the intended shape of the splint, “Cut or Punch” under the tool bar was used in the trimming process.

After the broad outline has been initially trimmed, the part must be duplicated for any further changes. The duplicate model is given a different color to aid in differentiation in later phases of the procedure.

The duplicate model is then offset by the required splint thickness of 3 mm (input via the pop-up window), resulting in a version 3 mm larger than the original to represent the final splint thickness (Figure 2d).

A hollow splint shell is created by subtracting the original model from the duplicate (offset by 3 mm), producing the desired 3 mm wall thickness (Figure 2e). The model is then further refined by applying the “Cut or Punch” function as previously mentioned. This stage guarantees that the model is cut and shaped in compliance with the cock-up design specification for splints.

**Figure 3:**
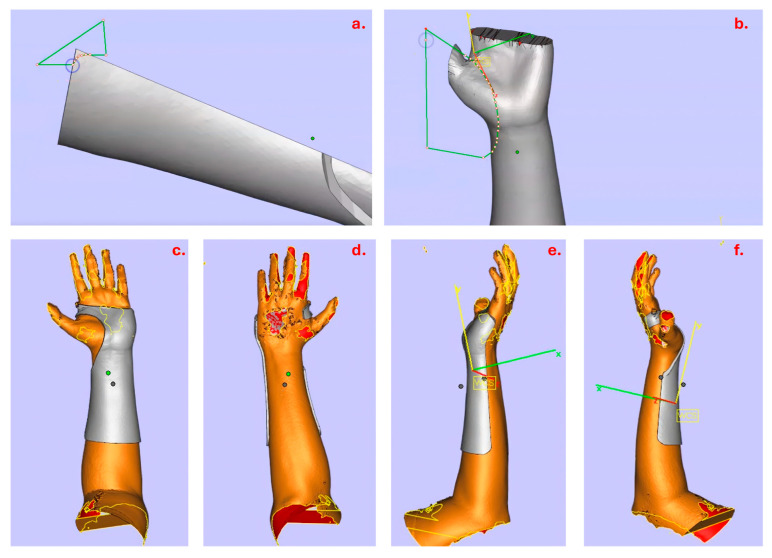
Edge trimming and finalized 3D model views. (**a**) Demonstration of round-edge generation with “Cut and Punch”, 1-cut applies to both sides; (**b**) demonstration of trimming at the thenar crease; (**c**–**f**) the finalized 3D model with estimated views: (**c**) volar view; (**d**) dorsal view; (**e**) medial view; (**f**) lateral view.

The last step of 3D sketching to refine the model is generating rounded edges and tailoring the splint model (Figure 3a). The cock-up splint should be tailored with consideration of the related bony prominences. The splint model is orientated to the corresponding plane and round edges are outlined using the “Cut or Punch” function as mentioned above. For the round edge at the dorsum, this requires rotating the model to various angles and trimming to prevent removing both palmar and dorsal surface at the same time by a single cut. The edge of the splint pattern is outlined at the flexion crease (underneath the distal palmar crease) and thenar crease (but not covering it) and extended down below the radial styloid process (Figure 3b). The thumb must not be obstructed as it abducts or attempts opposition. For the dorsum, it is trimmed at the proximal part to the head of the metacarpal of the index finger [22,23]. Finalized 3D models from in different views are shown in Figure 3 (Figure 3c–f).

### 2.4. 3D Printing and Polishing

**Figure 4:**
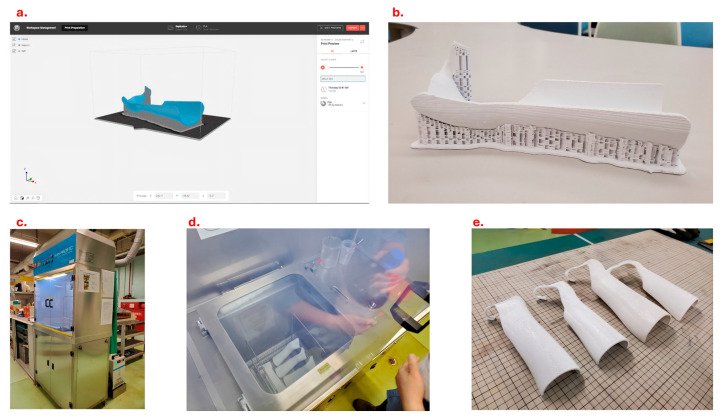
3D printing and vapor polishing. (**a**) The finalized model for 3D printing in software of MakerBot Replicator+ 3D printer; (**b**) image of the printed cock-up splint before polishing; (**c**) vapor polishing apparatus; (**d**) vapor polishing process; (**e**) 3D-printed splints after vapor polishing.

The finalized 3D models were printed in PLA using the 3D printer MakerBot Replicator+ (MakerBot, Sydney, Ausdtralia) with an enlarged scale of 107%, infill density of 20%, three shells and a layer resolution of 0.2 mm. The configuration of support adopted a breakaway configuration with a support angle of 40° (Figure 4a). The supporting parts of the printed models were then removed with cutting pliers and a graver. Sandpaper, mini files and a nail drill were applied to improve the roughness. It took 20–30 min to complete the process (Figure 4b). To further improve the surface of the 3D-printed splint, vapor polishing was adopted (Figure 4c,d). It was effective in obtaining a smoother surface by immersion with the chemical treatment using the Weldon 4 solvent with boiling water (100 °C) [24]. A standard procedure can be completed within 10 min, followed by 2 h drying. Finally, the 3D-printed splints after vapor polishing are shown in Figure 4 (Figure 4e). Consistent with the conventional approach, it is required to stick Velcro straps at the metacarpophalangeal joint, wrist and forearm levels. Clinicians may also add foam padding and slightly flare the bottom edge with a heat gun when necessary.

### 2.5. Participant Inclusion and Splint Fitting Assessment

Five participants were recruited to participate in the study using convenience sampling. The inclusion criteria included at least 18 years old and capable of reading and filling out a questionnaire in English. The exclusion criterion was having a physical impairment or limitation in activity in the non-dominant hand. A 3D-printed wrist splint was made for each participant using the methods described above. Participants were required to wear the splint for 7 h every day for 1 week before filling the Quest 2.0 form.

### 2.6. Outcome Measures for Feasibility

The splint-wearing experience is assessed by the Quebec User Evaluation of Satisfaction with Assistive Technology (QUEST 2.0). QUEST 2.0 is used as a standardized assessment tool for evaluating various assistive technology from the user’s perspective in clinical settings. Its content validity is achieved by an expert panel. It has an acceptable level of test–retest reliability as the intraclass correlation coefficient is above 0.75. It also has a moderate-to-excellent internal consistency as the Alpha values are greater than 0.7 and less than 0.9 [25].

QUEST 2.0 is a self-report questionnaire consisting of 12 questions related to the device and service delivery process. Users are asked to give a rating to each item using a 5-point scale. “1—not satisfied at all” is the lowest satisfaction level while “5—very satisfied” is the highest satisfaction level [25]. The higher the score, the more satisfied the user is with the assistive technology. In addition, the user is asked to select three most important items for them. Apart from quantitative data, QUEST 2.0 also collects qualitative data by asking users to offer comments on the assistive device and services. Also, an experienced occupational therapist in Hong Kong was invited to offer opinions on the splint qualities, technical errors, complications and other areas [25,26].

### 2.7. Data Collection and Analysis

Each participant filled in the QUEST 2.0 questionnaire upon the completion of splint wearing. There are 8 items under the Device subscale, including comfort, weight, durability, adjustments, simplicity of use, dimensions, effectiveness and safety; professional service, delivery, follow-up and repairs and servicing are the items under the Service subscale. The calculation of scores is structured as follows: the Device Subscale Score is calculated by dividing the total number of questions answered by the sum of the ratings for all device-related items. In a similar manner, the total scores for all service-related items are divided by the total number of questions completed to determine the Service Subscale Score. The sum of all item ratings, divided by the total number of questions answered, yields the Total QUEST Score. The total of all respondents’ Device Subscale Scores divided by five is the General Satisfaction Level (Device), which is used to assess overall satisfaction levels. Similarly, the General Satisfaction Level (Service) is calculated by adding up each participant’s Service Subscale Score and dividing the total by 5. Finally, the General Satisfaction Level to Assistive Technology is computed as the sum of the Total QUEST Scores across all participants divided by 5. The data was calculated and analyzed using Microsoft Excel, since only descriptive statistics were used. In addition, an experienced occupational therapist in Hong Kong was invited to offer opinions on the splints. A 3D-printed splint was delivered to the expert in-person. The expert then filled in the evaluation form (see Appendix A) after examination of the 3D-printed splint. Ethical approval of the study was granted by the Human Research Ethics Subcommittee at The Hong Kong Polytechnic University.

Ethical approval of the study was granted by the Institutional Review Board of The Hong Kong Polytechnic University.

## 3. Results

### 3.1. Quantitative Data from QUEST 2.0

All five participants completed the 7-day wearing protocol and experienced various activities during the period (see Appendix B). The satisfaction level of participants with the device and service based on QUEST 2.0 are shown in Table 2. In general, the average Total QUEST Score was 4.14 out of 5, while the range of Total QUEST Score given by each participant was between 3.75 and 4.42.

Under the Device subscale, the average scores of dimensions, weight, adjustments, safety, durability, ease of use, comfort, and effectiveness were 4, 4.6, 4.4, 4.2, 3.6, 4.6, 3.6 and 3.6 respectively. Under the Service subscale, the average score of service delivery, repairs/servicing, professional service and follow-up services were 4.4, 4, 4.6 and 4 respectively. Among the 12 items, the items with the highest rating were weight, ease of use and professional service at a rating of 4.6 out of 5. In contrast, the items with the lowest rating were durability, comfort and effectiveness at a rating of 3.6 out of 5. Two participants offered a rating of 2 to durability, while one participant gave a rating of 2 to effectiveness.

### 3.2. Qualitative Data from QUEST 2.0

The comments of users on the assistive device and services are shown in Table 3. Regarding dimensions, participants suggested enlarging the splint to better fit users and alleviate pressure points, particularly near the second metacarpophalangeal joint and proximal edge. Although the splint’s weight was not very light, it was not uncomfortable to wear. Velcro was thought to make adjusting the splint simple. Most participants reported that it was safe to use, especially when participating in sports; nonetheless, issues were brought up about sharp edges, breakage risk, and low wrist adhesion unless worn tightly. Perceptions of fragility and warping with extended usage were among the durability difficulties. Although the splint was typically easy to wear, it was somewhat inconvenient for everyday tasks like carrying goods. Hard materials, sharp edges, and inadequate airflow all affected comfort, causing skin discomfort and a stuffy sensation. Participants’ opinions on its effectiveness varied; some said it worked well as a cock-up splint, while others pointed out that the wrist shaping was inadequate for bigger movements. Finally, the scanning procedure under service delivery was laborious at first but became better with each attempt, while the long printing time remained a challenge.

According to the top three important factors perceived by each participant, which are shown in Table 4, durability was thought to be an important issue in four cases (Cases 1, 3, 4, and 5), emphasizing the need for improvements in the splint’s structural integrity. Comfort was a significant factor in all cases except Case 5, where weight was noted instead. Effectiveness was prioritized in Cases 1 and 2, while safety was a concern in Case 3. Dimensional issues emerged in Cases 4 and 5, suggesting the need for better fitting and sizing adjustments. Additionally, Case 1 uniquely highlighted the importance of professional service in the splint’s development and delivery process. This distribution of feedback underscores the multidimensional considerations necessary for optimizing the splint’s design and functionality. While participant feedback provided valuable user-centered insights into daily wear experience, professional evaluation from an experienced occupational therapist offered complementary clinical perspectives on splint design, functionality, and potential complications.

### 3.3. Expert Review

The expert opinion on the 3D-printed splint is summarized in Table 5. Regarding splint qualities, the expert indicated that the dimensions of the splint, which include a “U”-shaped height, edges that match the thickness of the forearm, and an ideal length that covers two-thirds of the forearm, are intended to improve fit and comfort. Although the brittle material of the splint lacks elasticity, it is substantially lighter than conventional ones. There have been no reports of itching or rash, and its clinical use is still in line with that of standard splints in terms of comfort and safety. Under technical errors and complications, issues like splint length relative to anatomic landmarks, range of motion limitations, and circumferential design are addressed with suggestions to widen the area near the radial styloid and improve surface contouring for better support and functionality. Lastly, other considerations include offering a variety of colors beyond traditional tones, reducing fabrication time and cost, and enabling remote fabrication for bed-bound patients, which enhances accessibility for individuals with limited mobility. These factors collectively aim to improve the splint’s usability, comfort, and accessibility.

## 4. Discussion

### 4.1. General Evaluation of 3D-Printed Splint

By using the aforementioned method, it is feasible to produce a 3D-printed splint that users could be satisfied with. The overall result of the QUEST 2.0 showed that the users were quite satisfied with the 3D-printed splint and the service received. A systematic review of splints fabricated via the conventional approach revealed that the ranges of average user satisfaction with the splint measured by the QUEST 2.0 Device subscale, Service subscale and Total Score were 3.7–4.53, 3.7–4.71 and 3.7–4.61 respectively [27]. This study is within the ranges of these three scales, suggesting that the user satisfaction level with the 3D-printed splint is comparable to the one produced via the conventional approach.

### 4.2. Clinical Implications: Pros of the 3D-Printed Splint

Considering the splint quality, weight and easiness of using the splint received the most positive feedback from the users and expert. Users were either quite satisfied or very satisfied with these two aspects. Meanwhile, the expert stated that the 3D-printed splint is lighter than the conventional one. The design of the 3D-printed splint is the same as the one made via the conventional approach for preserving its easiness of usage. This supports the proposition of current studies of the lightness of 3D-printed splints being more advantageous to users, compared with splints fabricated by the conventional approach [28]. In addition, all users were at least quite satisfied with the easiness in adjustments to the 3D-printed splint with the utilization of Velcro.

When it comes to the service provision to the users for producing the 3D-printed splint, professional service was the most appreciated aspects of the 3D-printed splints among all users. This suggests that clinicians could make good use of their expertise by offering information and attention to users. In addition, users were also at least quite satisfied with the service delivery. An efficient 3D scanning process could be accomplished after a few trials. This study reveals that some users would consider weight and professional service as part of the top three most important factors of getting and wearing the 3D-printed splint. With reference to the positive feedback on these areas by users, it was suggested that clinicians could independently produce a 3D-printed splint in a professional manner whose lightness meets users’ expectations. It is crucial to promote the user’s compliance with wearing the splint.

### 4.3. Clinical Implications: Areas of Improvements in 3D-Printed Splint

From the results, participants had lower satisfaction levels in the areas of durability, comfort and effectiveness among all items. The issues of brittleness and roughness were also highlighted in the expert review. These indicate that there is room for improvement of such elements of the 3D-printed cock-up splint.

#### 4.3.1. Durability and Brittleness

PLA is widely used in 3D-printed orthoses due to its biocompatibility, low cost, and favorable tensile/flexural strength (typically 50–70 MPa in optimized FDM prints), often exceeding traditional plaster in short-term load bearing [16,29]. However, its low impact resistance and elasticity result in brittleness, as evidenced here by cracking during support removal or accidental drops and perceived warping/deformation during wear (likely from overtightening or body heat/moisture) [17,18]. In this study, ~40% of early-batch splints (2/5 in one batch) showed fragility, attributed to printer calibration inconsistencies, infill pattern (linear vs. recommended honeycomb for shear strength), and lack of post-annealing. Recent work confirms that PLA orthoses can fail under repeated flexion or aging conditions [17,29]. Alternatives like PETG offer better ductility and fatigue resistance for clinical durability [17,18].

Nonetheless, the durability of PLA suggested by previous research is not supported by this study. A total of 11 3D-printed splints were fabricated in the four batches produced by different 3D printers in the same model (see Appendix C). Variations in the color scale and printing quality were found among the batches. In one of the batches, two out of five splints were found to be broken easily during the process of removing the supporting. Importantly, both splints exhibited pre-existing shallow cracks or missing filaments, and they were fabricated using separate 3D printers.. One of the splints was split into two pieces and broke into another piece after hitting the floor accidentally, while the other one was broken at the dorsum part of the splint. The splint was determined to be brittle, attributable to the material’s limited elastic properties and its insufficient capacity to resist external mechanical stresses, such as overtightening or high compressive forces. These affect the durability of the splint in clinical use. Meanwhile, the flat printing orientation and 0° raster angle applied in this study provided maximum tensile strength [30]. Other factors may also cause to the limited durability and brittleness observed in the splint. Accurate calibration is essential in fused deposition modeling (FDM) to maintain proper nozzle–bed distance, consistent material extrusion, precise filament deposition, and uniform void distribution across layers [31,32,33]. Recalibration should be performed as required to sustain optimal printing conditions, and consecutive prints produced on well-calibrated machines are expected to yield greater reproducibility and structural integrity. Variability among previous studies may be attributable to differences in infill geometry, printing patterns, and inconsistencies in printer performance; for example, honeycomb infill has been reported to enhance peak force and shear strength [34], while the development of advanced materials with improved load-bearing capacity and elasticity may further optimize mechanical performance.

#### 4.3.2. Low Breathability

As the splint model design in this study did not include any ventilation holes, it has relatively low breathability and therefore caused trapping of sweat, which was less comfortable for users. To facilitate moisture release and provide sufficient support for immobilization and strength of the splint, the lattice structure could be created with an optimized combination in the 3D printing setting: square pattern, printed with a nozzle diameter of 0.8 mm and tested at a strain rate of 5 mm/min [35].

#### 4.3.3. Improper Fit

The dimensions of the splints were suggested to be modified by enlarging the space between the palmar and dorsal side of the hand and the part in contact with the muscle bulk, as well as widening the space for the radial styloid. These could help reduce the unnecessary pressure points, better protect the wrist and gain greater satisfaction in comfort and effectiveness for users. For further improvement, the splint model could be divided into three proportions (i.e., hand, wrist to 1/3 length of forearm and the muscle bulk) and the scale of the parts could be enlarged accordingly during the 3D sketching process.

#### 4.3.4. Rough Edge and Surface

Previous research revealed a close relationship between the surface roughness and the mechanical properties as printed in the parallel direction [36]. Still, some rough parts in the 3D-printed splints were noticed in this study (i.e., both on the inner and outer surface of the splint).

Variations in print quality were observed, potentially attributable to inadequate calibration and inconsistent performance across the 3D printers used at the center. The surface and edge were evaluated to be rough even after the filing and vapor polishing process, which would reduce the level of comfort, willingness and compliance for clients to wear the splintage. To improve the smoothness of surface and outlook and protect users from skin lesions, the 3D printers are suggested to be calibrated regularly as to maintain the standard of printing quality, and a more advanced model of 3D printer may be considered when necessary. Meanwhile, the edges in the printed model could be created with a greater degree of round shape to further ensure safety of use.

### 4.4. Evaluation of the 3D Scanning Process

The 3D scanning process in the current study took around 5–10 min per participant depending on the stability of the participant and the proficiency of the operator. The presence of scan flaws was the major issue found during the scanning process. In the current study, the thumb web presented as the main area with missing parts (holes/artifacts), which could pose problems in the subsequent 3D sketching/modeling process. Rescanning is required to obtain a better scanned model; this could be intolerable for patients in clinical settings who are not able to maintain a posture for long. It is also important to address the proficiency of the operator in using the 3D scanner, as it can greatly reduce the time that patients need to maintain a posture for the scanning. The time required to be proficient at the 3D scanning process was not demanding as reflected in the current study; it took two trials (scanned five hands in each trial) for the operator to be acquainted with the process.

### 4.5. Evaluation of the 3D Sketching/Modeling Process

The 3D drawing process in the current study took around 15–30 min per splint depending on the design of the splint and the number of scan flaws. Compared with previous studies, in which the 3D modeling process for a hand orthosis required up to 2 h [9], the approach employed in the present study appears to offer potential time savings and greater feasibility for adoption in routine clinical practice. The 3D sketching/modeling process in the current study allowed for fixing small scan flaws that are commonly seen in the 3D scanning process. The 3D model is generated based on the original scanned model by offsetting its duplicate and reserves most of the properties of the scanned model, making the final 3D-printed splint better fit the users. The proposed methods also permit the operator to trim and refine the 3D model to have different shapes and round edges that are necessary for designing individualized splints.

### 4.6. Evaluation of the 3D Printing Process

The total printing time takes around 9–19 h per splint depending on the size of the splint (see Appendix C), substantially longer than conventional thermoplastic fabrication (~30–60 min). This drawback may be mitigated by faster printers, optimized parameters (e.g., higher layer height, lower infill), or batch production. The procedure of removing supporting parts of the printed model is around 20 to 30 min depending on the skillfulness of the clinician. It is important to note that the removal of supporting parts requires the manipulation of tools, such as cutting pliers and nail drills, which should be used with caution. As the surface of the printed model is rough, vapor polishing and padding are unavoidable afterwards, and add extra time and cost to the whole process. Dimensional accuracy and geometry reproduction were not quantitatively assessed (e.g., via coordinate measuring or rescanning), representing a limitation. Qualitative feedback indicated fit issues (e.g., pressure at metacarpophalangeal joint, radial styloid), suggesting that minor scaling offsets or contour refinements during modeling could improve conformity. Future studies should incorporate metrology for reproducibility.

### 4.7. Applicability in Clinical Practice and Recommendations

The methods outlined in this study are intended to provide clinicians with a detailed workflow for fabricating 3D-printed splints, rather than a prescriptive step-by-step manual. The approach does not require extensive prior expertise in 3D technologies, as portable scanners enable bedside acquisition for bed-bound patients, with modeling performed remotely if necessary. Nonetheless, clinicians are advised to familiarize themselves with the scanning process and conduct preliminary trials on healthy participants before applying the technique in patient care, as this can reduce scanning time and improve posture maintenance. Practical considerations include patient comfort during scanning (e.g., provision of sunglasses for light sensitivity), caution when applying positioning targets to compromised skin, and decision-making during digital modeling regarding areas to retain or remove. Once clinicians are proficient with the software, the workflow may be adapted to design other splint types, and protocols could be developed to allow occupational therapy assistants to assist in the process. While the manpower required can be minimized if the operator is experienced, financial constraints remain significant, as the scanner, printer, and software licensing entail substantial costs, and local access to finishing techniques such as vapor polishing is limited. Thus, clinical implementation requires both technical familiarity and adequate resources.

### 4.8. Implications and Limitations

This study presents a proof-of-concept workflow for fabricating cock-up splints using 3D scanning and printing technologies, offering a general clinician guideline rather than a definitive manufacturing protocol. The findings demonstrate feasibility but are limited to cooperative healthy participants capable of maintaining posture during scanning. Application to patient populations may present challenges, particularly in cases of hand deformities (e.g., rheumatoid arthritis, hypertonia), where scan quality and reproducibility could be compromised. Furthermore, the material employed in this study exhibited limitations in durability and comfort, underscoring the need for further material development. Future work should involve patient cohorts, systematic reproducibility testing, and exploration of alternative materials to establish clinical reliability and broaden applicability.

## 5. Conclusions

This proof-of-concept study demonstrates a feasible, clinician-led protocol for 3D-scanned and -printed cock-up wrist splints using PLA, with user satisfaction comparable to conventional methods and advantages in weight and customization. The detailed workflow requires basic computer techniques and offers a foundation for adoption. However, PLA’s brittleness, limited breathability, rough surfaces, and extended production times highlight material and process constraints requiring optimization (e.g., alternative filaments, ventilation designs, calibration protocols). Future work in patient cohorts and quantitative mechanical testing is essential before scalable clinical implementation.

## Figures and Tables

**Table 1:** Properties of the 3D splint manufacturing process.

Surface scanner	Ceaform GO!SCAN 50 (Creaform, Berwyn, PA, USA)
Scanner software	Ceamform VXelements 4.1 (Creaform, Berwyn, PA, USA)
3D design software	Magic® (Materialise, Leuven Belgium)
3D printer	MakerBot Replicator+ (MakerBot Industries, Brooklyn, NY, USA)
Extruder type	Smart Extruder+
Printing material	Polylactic acid (PLA)
Printing technique	Fused deposition modelling (FDM)
Layer thickness	Linear
Post-processing	0.2 mm
Favourable mechanical properties of printed splints	Rigid, lightweight, with limited thermoplasticity (adjustability), biodegradable

**Table 2:** Participants’ satisfaction level with the 3D-printed splint based on QUEST 2.0.

	Case 1	Case 2	Case 3	Case 4	Case 5	Total Score	Average
**Device**							
**Dimensions**	4	4	5	3	4	20	4
**Weight**	4	5	5	5	4	23	4.6
**Ease in Adjustments**	5	4	4	4	5	22	4.4
**Safety**	4	5	3	5	4	21	4.2
**Durability**	2	5	2	4	5	18	3.6
**Ease of Use**	5	4	4	5	5	23	4.6
**Comfort**	3	3	4	4	4	18	3.6
**Effectiveness**	2	4	4	4	4	18	3.6
**Device Subscale Score**	3.625	4.25	3.875	4.25	4.375		4.075
**Service**							
**Professional Service**	5	5	4	4	5	23	4.6
**Service Delivery**	5	4	4	5	4	22	4.4
**Follow-up Services**	N/A	5	3	4	N/A	12	4
**Repairing Service**	N/A	5	3	4	N/A	12	4
**Service Subscale Score**	5	4.75	3.5	4.25	4.5		4.4
**Total Score**	39	53	45	51	44		46.4
**Average Total QUEST Score**	3.9	4.42	3.75	4.25	4.4		4.14

**Table 3:** Comments on 3D-printed splint based on QUEST 2.0.

Items	Comments
Dimensions	Two participants suggested enlarging the size of the splint (i.e., the part in contact with the muscle bulk and the space of palm thickness) as to better fit users and relieve the pressure point below the 2nd metacarpophalangeal joint at the dorsum and the proximal edge of the 3D splint.
Weight	One participant reflected that the 3D splint was not very light but at least did not make her aware of extra weight on her arm while wearing.
Adjustment	Participants found the 3D splint easy to adjust with Velcro.
Safety	Most of the participants were satisfied with the splint as it was safe to use and did not come off even when playing sports; one participant felt that the splint was easy to break and had a relatively sharp edge; another participant felt that the 3D splint did not cling to her wrist well and she was only able to hold her wrist position when wearing it very tightly.
Durability	One participant mentioned that the 3D splint seemed to warp in the last two days of her wearing experience period and suspected that the deformation may be due to overtightening; another participant commented that the 3D splint looked easy to break.
Ease of use	One participant reflected that the 3D splint was easy to use in general, but it did cause some inconvenience during the splint-wearing period (e.g., carrying/picking up things).
Comfort	Participants reported that some edges hurt the skin when wearing the 3D splint for a long time; the edge at the palm/dorsum was sharp; one participant commented that the 3D splint material was quite hard and created some scratches on the skin surface; three participants felt stifled with the 3D splint as there were no pores and moisture was trapped inside.
Effectiveness	One participant felt that the splint did not mold properly around her wrist when carrying out larger movements; two participants evaluated that the splint served its purpose as a cock-up splint; another participant thought that the 3D splint was very strong.
Service delivery	One participant mentioned that it took a long time to scan the hand at first, but it became faster in the second round; another participant commented that scanning and getting the model was, but there were long printing hours.

**Table 4:** Top three important items selected by each case without prioritization.

Case 1	Case 2	Case 3	Case 4	Case 5
Durability	Professional service	Durability	Durability	Durability
Comfort	Comfort	Comfort	Comfort	Weight
Effectiveness	Effectiveness	Safety	Dimension	Dimension

**Table 5:** Expert review from experienced occupational therapist (Ms. Joy LAU Chung Man).

Aspect	Expert Comments/Observations
Splint Qualities (some elements adopted from QUEST 2.0) Dimensions (size, height, length, width) Weight Durability Comfort Safety Effectiveness Simplicity to use	The height of the splint can be taller so that the shape can build up a “U”. The edge is same as the forearm thickness. The length is optimal at 2/3 of forearm length. The weight is light, much lighter than the traditional splint. The material is not elastic enough and it’s very brittle. The material has the same comfort as the traditional one. No itchiness and rash after the splint application, so it is safe. Since the design is same as the traditional splint, the application is the same when applied in clinical use/testing.
Technical Errors and Complications Splint length relative to anatomic landmarks Range of motion limitations Circumferential nature Complications (e.g., swelling, numbness)	The part close to the radial styloid can be much wider to avoid the styloid touching the splint. It serves the function of restricting the wrist movements, similarly to the traditional one. The contour and outlook can be improved with smoothening the surface, and the base of the splint, which contacts the palmer side of the forearm, can increase the curve so as to improve the contact surface and provide more support.
Other	The color of the materials is more varied than the traditional one, which only has the options of skin tone or white. The fabrication time spent by the therapist can be reduced to reduce the cost of the splint fabrication. Since bed-bound patients cannot access the splint treatment, a remote version of splint fabrication can serve as another way to provide proper splint management for those cases with limited mobility.

## Data Availability

The original contributions presented in this study are included in the article. Further inquiries can be directed to the corresponding author.

## Appendix A. Evaluation Form for Expert Review

Name of Expert:

Splint Qualities (some elements adopted from QUEST 2.0) - Dimensions (size, height, length, width) - Weight - Durability - Comfort - Safety - Effectiveness - Simplicity to use
Technical Errors and Complications - Splint length relative to anatomic landmarks - Range of motion limitations - Circumferential nature - Complications (e.g., swelling, numbness)

## Appendix B. An Overview of Activities Participated in by Participants During the 7-Day Wearing Experience

	Participant 1	Participant 2	Participant 3	Participant 4	Participant 5
Activities (e.g., Self-care activity, shopping, doing housework, playing sports, using computer, etc.)	- Using a computer - Eating - Making handicrafts (kneading super-light clay) - Using a mobile phone - Taking public transport	- Self-care (using the toilet, hair brushing) - Using a tablet, mobile phone and computer - Attending f2f classes - Buying groceries - Having lunch (holding a bowl) - Transportation - Playing badminton (non-dominant hand)	- Self-care (grooming, hair brushing) - Using a computer and mobile phone - Attending online meetings and seminars - Having lunch - Gaming	- Self-care (grooming, cutting nails, using the toilet, tying ponytail) - Medication management - Using a computer - Attending f2f lecture - Taking a nap - Going outdoors - Holding an umbrella - Doing housework (washing dishes, drying and hanging clothes) - Dining and opening food delivery package - Grocery shopping and lifting bag - Filling 1 L water bottle - Taking photos with a mobile phone - Playing mobile phone games - Playing badminton (splint on non-dominant hand)	- Using computer - Dining - Using a mobile phone - Taking public transport - Shopping - Attending class
Feeling/comments after wearing (if applicable)	- A bit stifling - Shape not very fitting, could not cling to my wrist if not wearing it tightly, and would become loose if big movements made - Did not feel much stabbing/stinging pain from non-smooth parts, except the edge at the middle of the palm - In the last two days, the splint was a bit worn out and its shape changed a bit (due to my arm being pinched by the splint once) - Clear scar left on palm, back of hand and arm while taking off the splint - Inconvenience while doing handicrafts/using computer due to limited wrist angle and arm movement	- In general felt uncomfortable around the styloid process of the radius - Sweaty and stifling when wearing it outdoors (summer) and while/after playing badminton - Inconvenience when using the toilet	- In general no major hindrance to tasks - In general it fits the forearm and wrist comfortably - Sweating decreases its comfort, and slight allergic response found (may be due to hot weather) - Inconvenience when doing grooming, cleaning and cleansing tasks, which involve water - The surface may be rough, not flexible and cause sweating due to hot weather	- Slightly stressful to the palmar arch and wrist (dorsal), redness - Scar on wrist and muscle Traveling outdoors - Kept moisture from the first 10 min of wearing splint outdoors, sharp pain on dorsal part Playing badminton - Sweat became a lubricant, making the splint less tight and causing it to slip - Redness on palmar arch after playing badminton Cutting nails - Requires lifting up the hand a bit to adjust the angle for cutting Washing dishes - Easy to drain and dry the splint with paper towel - Added some stress to the palmar arch and the bony prominence when holding up heavy bowls or pans	- Inconvenient to carry bags or take objects - Not so appropriate in hotter environments - Moisture appears quickly after wearing - Good for resting in static position
Photos of hand/splint condition after wearing (if applicable)			No serious concerns		
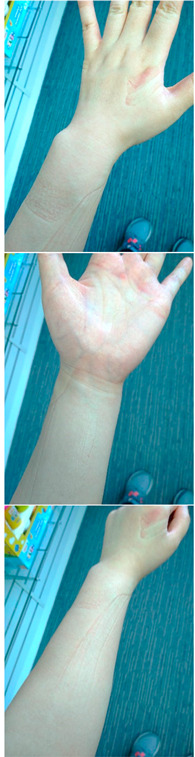
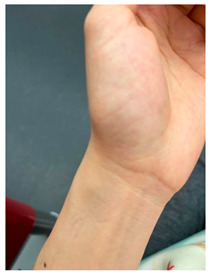
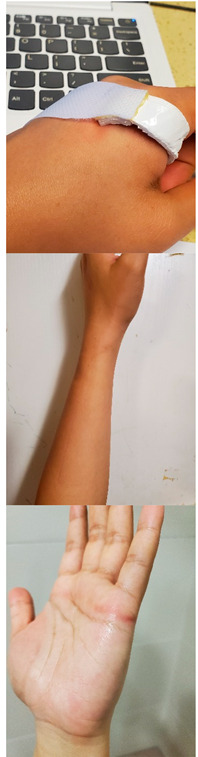
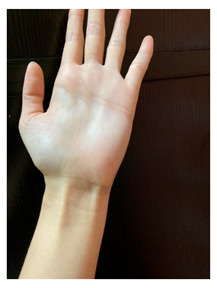

## Appendix C. Summary Table of the Four Batches’ Fabrication

	1st Batch	2nd Batch	3rd Batch	4th Batch
3D scanning				
Date	30 September 2021	19 May 2022	N/A	N/A
Time used	10–15 min per participant	5–10 min per participant	N/A	N/A
3D sketching				
Date	10 May 2022	30 May 2022	6 July 2022	N/A
Time used	About 40 min for 1 splint	About 15–30 min/splint	Refined 2 CADs within 10 min	N/A
3D printing				
Date	17 May 2022	2 June 2022	6 July 2022	8 July 2022
Time used	15 h 26 min (213.3 g for 2)	13 h 23 min (181 g for 1 ) 19 h 36 min (258.8 g for 2 ) 15 h 21 min (204.6 g for 2)	11 h 2 min (148.6 g for 1) 8 h 35 min (115.7 g for 1)	11 h 2 min (148.6 g for 1) 11 h 2 min (148.6 g for 1)
Remarks	Printed total of 2 splints in 1 printer	Printed total of 5 splints in 3 printers Broken in later process	Printed total of 2 splints in 2 printers	Printed total of 2 splints in 2 printers
min				
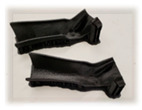
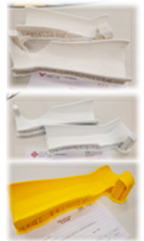
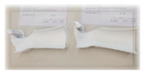
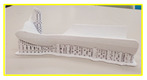
Removing supporting				
Date	19 May 2022	6 June 2022	8 July 2022	11 July 2022
Time used	15 min/splint	45 min/splint	27 min/splint	25 min/splint
Vapor polishing				
Date	N/A	7 June 2022	12 July 2022	
Time used	N/A	5 min for vapor polishing, 2 h for drying		
